# High-salt-driven gut microbiota dysfunction aggravates prostatitis by promoting AHR/SGK1/FOXO1 axis-mediated Th17 cell differentiation

**DOI:** 10.1186/s40779-025-00607-1

**Published:** 2025-05-19

**Authors:** Jing Chen, Rui Feng, Bin-Bin Gong, Wei-Kang Wu, Bang-Shun Dai, Rui Tan, Wen-Long Xu, Tong Meng, Xiao-Bin Wang, Yun-Zheng Xiao, Cheng Yang, Li Zhang, Chao-Zhao Liang

**Affiliations:** 1 Department of Urology, the First Affiliated Hospital of Anhui Medical University, Institute of Urology, and Anhui Province Key Laboratory of Urological and Andrological Diseases Research and Medical Transformation, Anhui Medical University, Hefei, 230022 China; 2 Department of Urology, Shuguang Hospital, Shanghai University of Traditional Chinese Medicine, Shanghai, 201203 China; 3 Department of Urology, Southern University of Science and Technology Hospital, Shenzhen, 518055 Guangdong China; 4 Center for Scientific Research of the First Affiliated Hospital of Anhui Medical University, Hefei, 230022 China

**Keywords:** Chronic prostatitis/chronic pelvic pain syndrome (CP/CPPS), High-salt diet, 5-Hydroxyindole acetic acid (5HIAA), Serum and glucocorticoid-regulated kinase 1 (SGK1), Th17 cells

## Abstract

**Background:**

Chronic prostatitis/chronic pelvic pain syndrome (CP/CPPS) is a frequently encountered disorder characterized by voiding symptoms and pelvic or perineal pain. Proinflammatory T helper 17 (Th17) cells are essential for triggering the development of CP/CPPS. High-salt diet (HSD) consumption has been found to cause an accumulation of sodium chloride in peripheral organs, inducing autoimmune responses via the Th17 cell axis. It is currently unknown whether HSD affects the etiology and course of CP/CPPS.

**Methods:**

Patients diagnosed with CP/CPPS were evaluated with the National Institutes of Health Chronic Prostatitis Symptom Index scoring system, and the correlation between the symptoms of CP/CPPS with HSD was analyzed. The experimental autoimmune prostatitis (EAP) mouse was established and the mice were fed either a normal-salt diet (NSD) or HSD for 6 weeks to investigate the impact of HSD on CP/CPPS. Then, 16S ribosomal RNA sequencing and untargeted metabolomics were introduced to detect the differences in the gut microflora composition and metabolite profiles between NSD-fed and HSD-fed mice, followed by fecal microbiota transplantation, 5-hydroxyindole acetic acid (5-HIAA) supplementation, aryl hydrocarbon receptor (AHR) inhibition, and in vitro Th17 differentiation experiments, which were performed to explore the mechanisms underlying HSD-aggravated CP/CPPS. Finally, chromatin immunoprecipitation assay and quantitative polymerase chain reaction were conducted to validate whether AHR can serve as a transcription factor by interacting with the serum and glucocorticoid-regulated kinase 1 (*Sgk1*) promoter in CD4^+^ T cells.

**Results:**

Increased salt consumption had a positive correlation with symptom scores of CP/CPPS patients, which was validated by feeding EAP mice with HSD, and HSD worsened the prostate inflammation and tactile allodynia in EAP mice through promoting the differentiation of CD4^+^ T cells to Th17 cells. HSD exacerbated EAP by significantly reducing the relative abundance of beneficial gut microflora, such as Lactobacillaceae, and gut microbiota metabolite 5-HIAA, which is related to tryptophan metabolism. The prostate inflammation, tactile allodynia, and proportion of Th17 cells in mice that received fecal suspensions from the EAP + HSD group were significantly more severe or higher than those in mice that received fecal suspensions from the EAP + NSD group. However, 5-HIAA supplementation ameliorated the symptoms of EAP caused by HSD through inhibiting the differentiation of CD4^+^ T cells to Th17 cells, while AHR inhibition abrogated the protective effects of 5-HIAA supplementation on EAP mice fed a HSD through promoting the differentiation of CD4^+^ T cells to Th17 cells. Mechanistically, it has been revealed that the SGK1/forkhead box protein O1 (FOXO1) pathway was significantly activated during cytokine-induced Th17 cell differentiation, and AHR has been shown to inhibit SGK1 transcription by interacting with the *Sgk1* promoter in CD4^+^ T cells to inhibit FOXO1 phosphorylation, consequently restoring the equilibrium of Th17 cell differentiation.

**Conclusion:**

Our findings indicated that high salt intake represented a risk factor for the development of CP/CPPS as it promoted the differentiation of CD4^+^ T cells to Th17 cells through the 5-HIAA/AHR/SGK1/FOXO1 axis, which might be a potential therapeutic target for CP/CPPS.

**Supplementary Information:**

The online version contains supplementary material available at 10.1186/s40779-025-00607-1.

## Background

Chronic prostatitis/chronic pelvic pain syndrome (CP/CPPS) affects 35–50% of men during their lifetime and is associated with pain and voiding difficulties [1]. An earlier investigation involving over 10,600 individuals revealed that the incidence of CP/CPPS symptoms was 8.2%. The cause of CP/CPPS is intricate and is not completely understood [2].

Inflammation-mediated abnormal pelvic floor neuromuscular activity, imbalanced neuroendocrine homeostasis, and disorganized lower urothelial cells, have been identified as potential risk factors for CP/CPPS [3, 4]. It is worth noting that cumulative evidence from human subjects and experimental animals hinted that this syndrome might be a result of dysregulated autoimmune responses against prostate antigens (PAgs) [5, 6]. T lymphocytes, particularly CD4^+^ T cells, are essential in the development of CP/CPPS, as revealed by a widely used experimental autoimmune prostatitis (EAP) mouse model [7, 8]. Furthermore, several studies have shown that immunization with PAgs would induce an immune response associated with increased differentiation of CD4^+^ T cells to abnormal T helper 1 (Th1) and T helper 17 (Th17) cells, thereby aggravating prostate inflammation and chronic pelvic pain [9, 10]. Correspondingly, blockade of interferon-γ (IFN-γ) or interleukin-17 (IL-17), the specific cytokines secreted by Th1 and Th17 cells, could significantly abolish the typical symptoms of CP/CPPS, further supporting the key roles of Th1 and Th17 cells [11, 12]. However, the mechanisms underlying the increased proportions of Th1 and Th17 cells during the development of CP/CPPS remain not entirely clear, there is an urgent need for in-depth exploration.

When it is consumed in proper amounts, sodium is a critical nutrient for the physiological function of living organisms that maintains normal osmotic pressure, pH, body fluid distribution, and most metabolic processes [13]. However, even though sodium chloride (NaCl) has an effect on the human tissue microenvironment, its critical role in controlling the immune system, allergies, and especially autoimmunity is occasionally neglected [14, 15]. Notably, consuming a high-salt diet (HSD) over time leads to the accumulation of NaCl, which has been identified as a potent factor associated with a strong inflammatory response. Emerging reports have revealed that HSD could aggravate autoimmune encephalomyelitis, ulcerative colitis, and cardiovascular diseases, by inducing the differentiation of CD4^+^ T cells to pathogenic Th17 cells [16–19] or the polarization of proinflammatory macrophages [20, 21]. Nevertheless, it is currently unknown whether HSD plays a crucial role in the pathogenesis and progression of CP/CPPS.

Despite considerable efforts, the etiology of CP/CPPS remains poorly understood, and effective intervention strategies are insufficient. Accordingly, this study aimed to explore the correlation between HSD and CP/CPPS, as well as the impact of HSD on the pathogenesis and progression of CP/CPPS, which may provide insights to design precision-based intervention strategies for CP/CPPS therapy.

## Materials and methods

### CP/CPPS patients and healthy men recruitment

The Ethics Committee of the First Affiliated Hospital of Anhui Medical University authorized a protocol for participant recruitment (PJ2024-03-37). Before any information was obtained, each participant provided informed consent.

The inclusion criteria for CP/CPPS patients are: (1) pelvic or perineal pain or discomfort with an NIH-CPSI score of > 4, and (2) symptoms lasting > 3 months [10]. The exclusion criteria are: (1) patients with various disorders of the urinary system, such as interstitial cystitis, urethritis, kidney and bladder stones, bacterial or acute prostatitis, urinary tuberculosis, and urinary tract infections; (2) patients with serum prostate-specific antigen concentration > 4.0 ng/ml; and (3) patients with prostate ultrasonography indicating residual urine volume ≥ 50 ml or prostate volume > 2 cm × 3 cm × 4 cm. Based on the above criteria, we collected demographic characteristics, dietary sodium intake data, and NIH-CPSI questionnaire scores from 710 healthy men and 434 CP/CPPS patients (Additional file 1: Table S1).

### Statistical calculation for the average daily intake of sodium

In accordance with the previous study, a customized questionnaire was applied to document the average daily intake of sodium [22], in which the sodium content per 100 g of commonly consumed foods was compiled to estimate the average daily intake of sodium, based on the Chinese Food Composition Tables [23].

### Animal studies

Nonobese male diabetic/LtJ (NOD) mice (*n* = 76) were obtained from the Nanjing University’s Nanjing Biomedical Research Institute in China [SCXK(Suzhou)2023-0009]. The animal investigations were carried out by strictly following the Guide for the Care and Use of Laboratory Animals at Anhui Medical University and approved by the Animal Care Committee of Anhui Medical University (LLSC20241761).

To establish the EAP mouse model, homogenized PAgs were mixed with an equal volume of complete Freund’s adjuvant (CFA), followed by repetitively subcutaneous injection to the base of the tail and hind footpad of NOD mice on day 0 and day 28 [12]. The animal experiments were conducted in 4 phases. In the first phase, mice were divided into 4 groups to detect the impact of HSD on the pathogenesis of EAP: normal mice fed a normal-salt diet (NSD) including ordinary chow and water (Ctrl + NSD, *n* = 4), normal mice fed a HSD with 4% NaCl in the chow and 1% NaCl in the water (Ctrl + HSD, *n* = 4), EAP mice fed a NSD (EAP + NSD, *n* = 4), and EAP mice fed a HSD (EAP + HSD, *n* = 4). In the second phase, mice were divided into 4 groups to determine whether alterations in gut microbiota were responsible for the exacerbating effect of HSD on EAP: pseudogerm-free mice, which were treated with a cocktail of antibiotics consisting of metronidazole (1 g/L, Sigma, USA), ampicillin (1 g/L, Sigma, USA), and neomycin sulfate (1 g/L, Sigma, USA) for 2 weeks [21], received fecal microbiota transplantation (FMT) with fecal samples collected from Ctrl + NSD mice (Ctrl-NSD-FMT, *n* = 4), pseudogerm-free mice received FMT with fecal samples collected from Ctrl + HSD mice (Ctrl-HSD-FMT, *n* = 4), pseudogerm-free mice received FMT with fecal samples collected from EAP + HSD mice (EAP-NSD-FMT, *n* = 4), and pseudogerm-free mice received FMT with fecal samples collected from EAP + HSD mice (EAP-HSD-FMT, *n* = 4). In the third phase, mice were divided into 5 groups to explore whether the decrease in 5-hydroxyindole acetic acid (5-HIAA) abundance participated in the exacerbating effect of HSD on EAP: Ctrl + NSD (*n* = 4), Ctrl + HSD (*n* = 4), EAP + NSD (*n* = 4), EAP + HSD (*n* = 4), and EAP + HSD co-administrated with 5-HIAA (25 mg/kg body weight per day, EAP + HSD + 5-HIAA, *n* = 4). In the fourth phase, mice were divided into 6 groups to further explore whether the protective effect of 5-HIAA supplementation in EAP + HSD mice was dependent on AHR activity: Ctrl + NSD (*n* = 4), Ctrl + HSD (*n* = 4), EAP + NSD (*n* = 4), EAP + HSD (*n* = 4), EAP + HSD + 5-HIAA (*n* = 4), and EAP + HSD + 5-HIAA co-administrated with the aryl hydrocarbon receptor (AHR) inhibitor CH223191 (CH, 10 mg/kg body weight per day, EAP + HSD + 5-HIAA + CH, *n* = 4).

### Materials and reagents

The following reagents and antibodies were used: CFA (#F5881, Sigma, USA); commercial enzyme-linked immunosorbent assay (ELISA) kits for IFN-γ (#E-EL-M0048c), IL-1β (#E-EL-M0037), IL-17A (#E-EL-M0047c), tumor necrosis factor-α (TNF-α, #E-EL-M3063), and 5-HIAA (#E-EL-0075c) (Elabscience, China); anti-mouse CD4-fluorescein isothiocyanate (FITC, #553047), CD3e-allophycocyanin (APC, #561826), IFN-γ-phycoerythrin (PE, #562020), IL-17-PE (#559502), anti-human CD8-FITC (#555634), CD3-APC (#555342), IL-17-PE (#560436) (BD, USA); anti-CD3e (#BE0001-1), anti-CD28 (#BE0015-1), anti-IFN-γ (#BE0055), anti-IL-4 (#BE0045) (Bio X Cell, USA); IL-23 (#CS31), transforming growth factor-β1 (TGF-β1, #CA59), IL-6 (#CG39) (Novoprotein, China); anti-AHR (1:1000, #NB100-128, Novus, USA); anti-SGK1 (1:1000, #AF6789), anti-FOXO1 (1:1000, #AF6416), anti-p-FOXO1 (1:1000, #AF3417), anti-retinoic acid receptor-related orphan receptor gamma-t (RORγt; 1:1000, #DF3196), and anti-GAPDH (1:5000, #AF7021) (Affinity, USA).

### Pelvic pain symptom evaluation

The day before the conclusion of the investigation, von Frey filaments were used to evaluate the mice for abnormal pain in the lower abdomen near the prostate. The mice were acclimatized to the environment for half an hour before testing. The examination of pain allodynia was performed by evaluating the tactile allodynia and hyperalgesia through the use of von Frey filaments with pressure from 0.04 to 4.0 g. A positive response to filament stimulation was classified as a sudden contraction of the abdominal area, rapid licking or scratching, or jumping off the stimulus site [4, 11, 12]. Finally, the percentage of positive responses was presented for evaluation.

### Hematoxylin–eosin (HE) staining

To obtain prostate tissues, the rodents were euthanized via neck dislocation after they were anesthetized. Pathological changes were identified via HE staining of prostate tissues. Following the sectioning of the lung tissues into 5-μm thick sections, they were stained with hematoxylin and eosin. After a standard dehydration process, sections of prostate tissue were sealed with neutral resin before examination with a light microscope. The inflammatory histopathological changes were graded on a scale of 0 to 3 as follows: 0, no inflammation; 1, mild but definite vascular hyperplasia with mononuclear cell infiltration in the interstitium; 2, moderate vascular hyperplasia and mononuclear cell infiltration in the interstitium; and 3, severe vascular hyperplasia, hemorrhage, and extensive mononuclear cells infiltration in the interstitium [24].

### ELISA

After the peripheral blood samples from each mouse were centrifuged at 800 × *g* for 20 min, the serum was obtained. The concentrations of IFN-γ, IL-1β, IL-17A, TNF-α, and 5-HIAA in mouse serum were quantified by using the commercial ELISA kits.

### Flow cytometry

Single-cell suspensions were prepared as previously described [4, 11]. A MoFlo XDP flow cytometer (Beckman Coulter, USA) was employed to separate Th17 and Th1 cells from the mouse spleen. The sorted cells were then subjected to treatment with anti-mouse CD4-FITC, IFN-γ-PE, CD3-APC, and IL-17-PE antibodies at ambient temperature for 1 h. The proportions of Th17 cells were analyzed using CD4-FITC, CD3-APC, and IL-17-PE antibodies. The proportions of Th1 cells were analyzed via the use of IFN-γ-PE, CD4-FITC, and CD3-APC antibodies.

### Isolation and differentiation of naïve CD4^+^ T cells in vitro

Naïve CD4^+^ T cells from mice and humans were obtained by using the mice naïve CD4^+^ T cells isolation kit (#130–104-453, Miltenyi Biotec, Germany) and the human naïve CD4^+^ T cells isolation kit (#130–094-131, Miltenyi Biotec, Germany), respectively. Naïve CD4^+^ T cells were cultured in 6-well plates which were precoated with anti-CD28 (10 μg/ml) and anti-CD3 (10 μg/ml). To stimulate the differentiation to Th17 cell, cytokines IL-6 (40 ng/ml), IL-23 (40 ng/ml), TGF-β (1 ng/ml), anti-IFN-γ (20 μg/ml), and anti-IL-4 (20 μg/ml) antibodies were added. The groups included: normal, stimulating factor, stimulating factor + HSD (40 mmol/L NaCl), stimulating factor + HSD (40 mmol/L NaCl) + 5-HIAA (10 mmol/L), and stimulating factor + HSD (40 mmol/L NaCl) + 5-HIAA (10 mmol/L) + CH (10 μmol/L). Flow cytometry was introduced to assess the differentiation ratio of Th17 cells.

### Chromatin immunoprecipitation (ChIP)-quantitative polymerase chain reaction (qPCR)

The ChIP procedure was conducted with the EZ ChIP kit (#17–371, Millipore, Germany). The primer sequences were listed in Additional file 1: Table S2. In summary, CD4^+^ T cells were chemically fixed with 1% formaldehyde for 10 min, and the resulting DNA fragments were acquired through ultrasonication. Afterward, the lysates were incubated in the presence of magnetic beads with either an anti-IgG or anti-AHR antibody (#GTX22769, GeneTex, USA). The obtained DNA samples were gathered and purified before analysis using qPCR. Please see details of Additional file 1: Methods for other methods.

### Statistical analysis

Each outcome is shown as the mean ± standard deviation (SD) or *M* (*Q*_1_, *Q*_3_). SPSS 21 software (IBM Corp., USA) was used for statistical analysis. Two-tailed Student’s *t*-tests or one-way analysis of variance (ANOVA) test followed by Bonferroni post hoc test and Wilcoxon rank sum test were used to assess group differences. *P*-values less than 0.05 are considered statistical significance.

## Results

### HSD was positively correlated with severe CP/CPPS symptoms

**Fig. 1:**
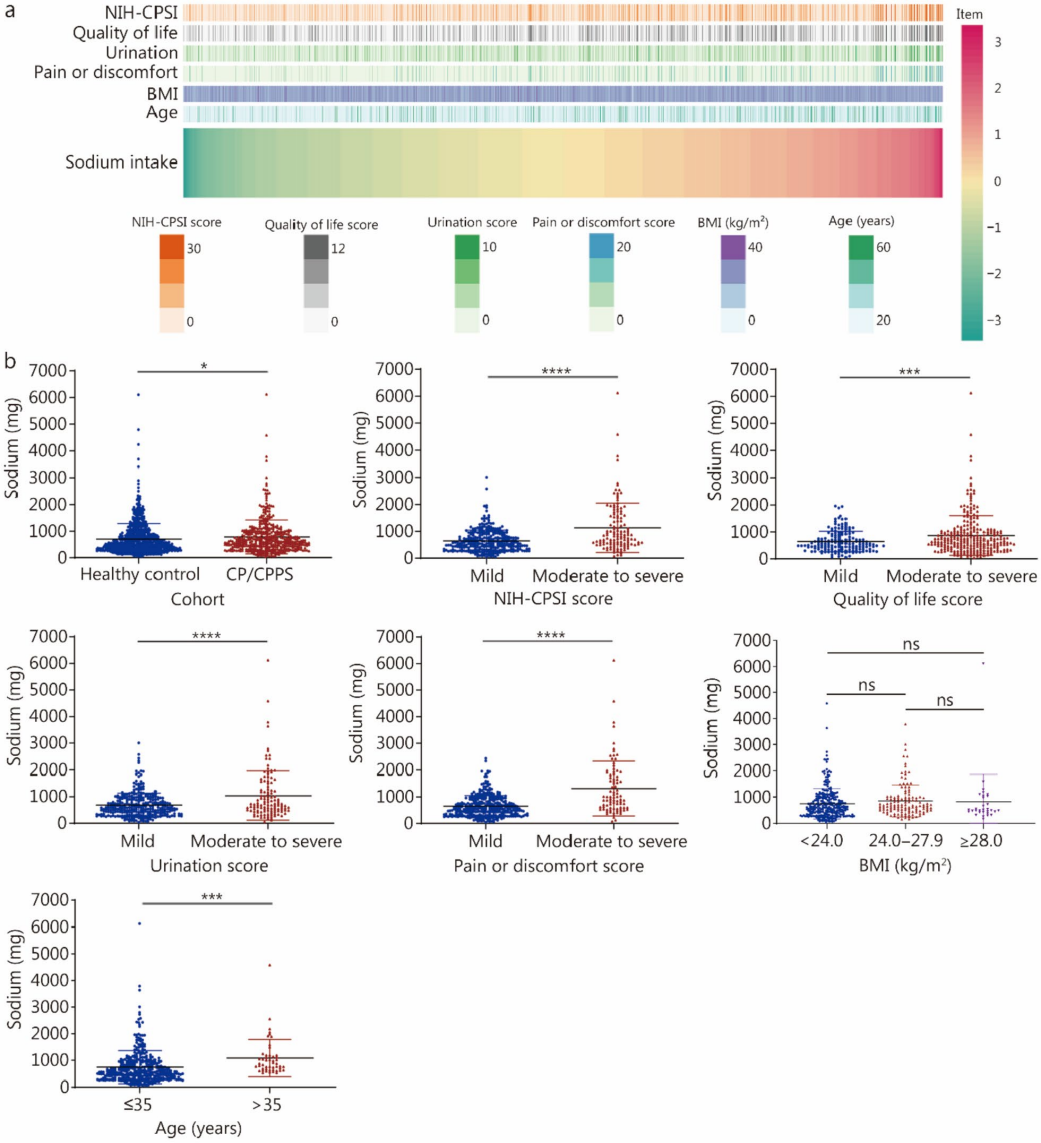
Severe CP/CPPS symptoms were positively correlated with HSD. **a** Heatmap illustrating the relationship between sodium consumption and clinical characteristics. **b** The sodium intake of CP/CPPS patients was greater than that of healthy controls. Sodium intake increased with NIH-CPSI scores, quality of life scores, urination score, pain or discomfort score, and age. ^*^P < 0.05, ^***^P < 0.001, ^****^P < 0.0001; ns non-significant. CP/CPPS chronic prostatitis/chronic pelvic pain syndrome, BMI body mass index, HSD high-salt diet

According to demographic characteristics, dietary sodium intake data, and NIH-CPSI questionnaire scores from participants, HSD was positively correlated with severe symptoms of CP/CPPS (*P* < 0.0001; Additional file 1: Table S3). CP/CPPS patients had considerably greater salt intake than healthy people, according to the distribution of sodium intake and clinical variables among patients and healthy individuals (Fig. 1a). Specifically, there was a positive association between sodium intake and the total NIH-CPSI score (*P* < 0.0001), quality of life score (*P* = 0.0004), urination score (*P* < 0.0001), and pain or discomfort score (*P* < 0.0001); moreover, a positive correlation was also observed between high sodium intake and increasing age (*P* = 0.0004; Fig. 1b, Additional file 1: Table S3). These results imply that CP/CPPS development may be influenced by HSD.

### HSD promoted Th17 cell development and worsened prostate inflammation in EAP mice

**Fig. 2:**
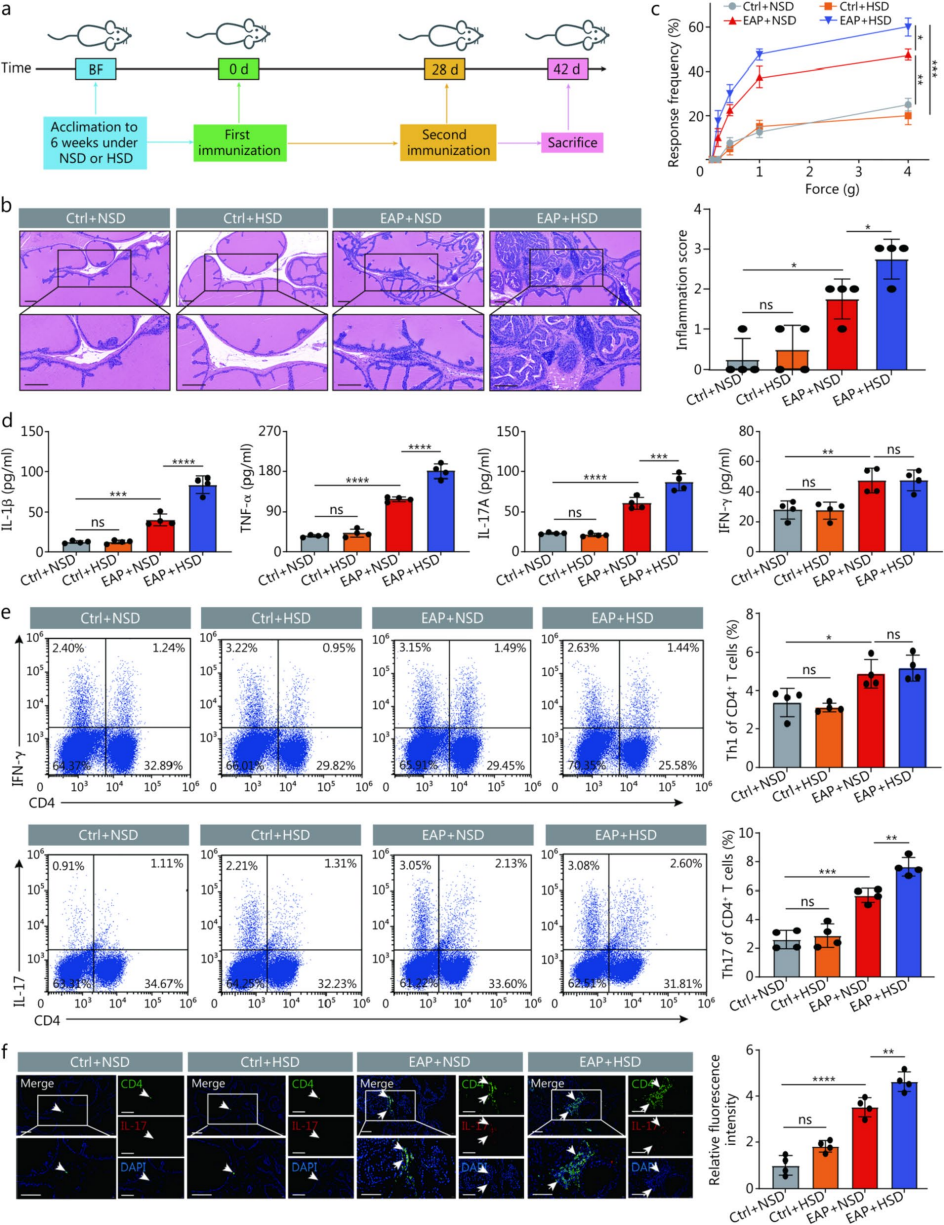
HSD promoted Th17 cell development and worsened prostate inflammation in EAP mice. **a** Simple schematic diagram of the implementation plan of the experiment. **b** HE staining and inflammation scores of prostate tissues (scale bar = 100 μm, *n* = 4). **c** Tactile allodynia development in the 4 groups of mice (*n* = 4). **d** The levels of IL-1β, TNF-α, IL-17A, and IFN-γ in the serum of the mice in the 4 groups (*n* = 4). **e** Proportion of Th1 and Th17 cells of CD4^+^ T cells detected by flow cytometry in the splenic lymphocytes of vaccinated mice from those 4 groups (*n* = 4). **f** The infiltration of Th17 cells in prostate tissues from mice was determined by immunofluorescence (white arrowheads, scale bar = 100 μm, *n* = 4). ^*^P < 0.05, ^**^P < 0.01, ^***^P < 0.001, ^****^P < 0.0001; ns non-significant. BF breeding feed, EAP experimental autoimmune prostatitis, HSD high-salt diet, NSD normal-salt diet, IL-1β interleukin-1β, TNF-α tumor necrosis factor-α, IFN-γ interferon-γ, IL-17A interleukin-17A, DAPI 4',6-diamidino-2-phenylindole, Th T helper

As mentioned above, long-term HSD consumption may worsen various diseases. We fed an HSD to EAP mice to verify its impact on the development of CP/CPPS (Fig. 2a). Mice administered HSD showed more severe edema, angiogenesis, and inflammatory cell infiltration in the prostate stroma relative to EAP mice fed an NSD (Fig. 2b). Additionally, at forces of 1.0 and 4.0 g, the frequency of tactile allodynia in EAP + HSD mice was consistently greater than that of EAP + NSD mice (Fig. 2c). Similarly, the IL-1β, TNF-α, and IL-17A concentrations in the serum of the EAP + HSD group were considerably elevated compared with those in the EAP + NSD group. Nevertheless, the IFN-γ levels did not differ significantly between the two groups (Fig. 2d). Since proinflammatory Th1 and Th17 cells are known to be involved in CP/CPPS, the impact of HSD on Th1 and Th17 cell differentiation was assessed. Our research revealed that the proportion of Th17 cells was greater in the mice in the EAP + HSD group than in the EAP + NSD group; however, no marked differences in the Th1 cell proportions were recorded between the two groups (Fig. 2e). Additionally, immunofluorescence staining of prostate tissue from the mice revealed that the infiltration of Th17 cells in the prostate tissues of the mice in the EAP + HSD group was significantly greater than that in the prostate tissues of the mice in the EAP + NSD group (Fig. 2f). These results suggested that HSD aggravated CP/CPPS by increasing the differentiation of CD4^+^ T cells to Th17 cells rather than Th1 cells.

### Gut microflora and metabolite profiles in HSD-fed EAP mice significantly differed from those in NSD-fed EAP mice

**Fig. 3:**
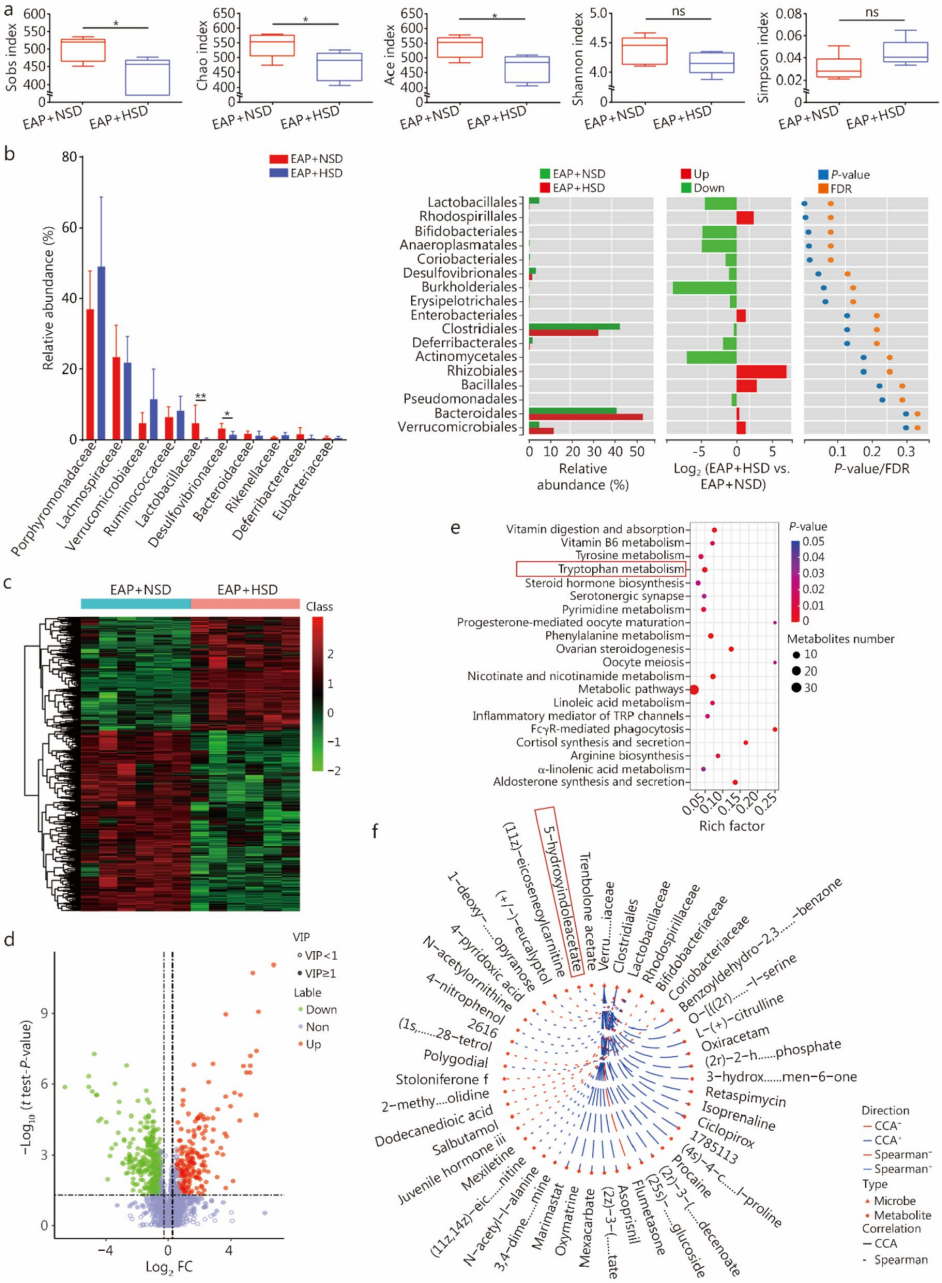
Metabolites and microflora compositions were dissimilar between EAP mice fed an HSD and an NSD. **a** Bacterial 16S rRNA fecal sample α-diversity indexes for mice in the EAP + HSD and EAP + NSD groups. **b** Comparison of the gut microflora composition between mice in the EAP + HSD and EAP + NSD groups. **c** Heatmap of metabolites in serum of mice in the EAP + HSD and EAP + NSD groups. **d** Metabolites that were differentially abundant in the serum of mice in the EAP + HSD or EAP + NSD group. **e** Kyoto Encyclopedia of Genes and Genomes (KEGG) analysis revealed enriched pathways associated with the differentially abundant metabolites of NOD mice between the EAP + HSD and EAP + NSD groups. The tryptophan metabolism abnormalities were indicated in red. **f** Integrated association analysis of the 16S rRNA and LC–MS data of the mice in the EAP + HSD and EAP + NSD groups. ^*^P < 0.05, ^**^P < 0.01; ns non-significant. NOD nonobese male diabetic/LtJ, EAP experimental autoimmune prostatitis, HSD high-salt diet, NSD normal-salt diet, LC–MS liquid chromatography-mass spectrometry, TRP tryptophan, FDR false discovery rate, CCA canonical correlation analysis

Serum and fecal samples were analyzed using liquid chromatography-mass spectrometry (LC–MS) and 16S ribosomal RNA (16S rRNA) sequencing to identify any potential alterations in the microbe and metabolite compositions to examine the effects of HSD on the development of CP/CPPS. The α-diversity indexes, such as the Sobs, Chao, and Ace indexes, were significantly lower in the EAP + HSD mice than in the EAP + NSD mice (Fig. 3a). These findings revealed that the α-diversity of the gut microbiota was significantly altered. When the EAP + HSD mice were compared with the EAP + NSD mice, the relative abundances of Lactobacillaceae and Desulfovibrionaceae were substantially lower (Fig. 3b). In addition, many differentially abundant metabolites were found between EAP + HSD mice and EAP + NSD mice (Fig. 3c, d). The metabolic pathway enrichment analysis revealed a high enrichment of the tryptophan metabolism pathway in the differentially abundant metabolites (Fig. 3e). We conducted a comprehensive correlation analysis with the data provided by 16S rRNA sequencing and LC–MS to elucidate the associations among microbe and metabolite abundances. Intriguingly, we observed that the decreased abundance of 5-HIAA, an important tryptophan metabolite, was closely related to the decrease in Lactobacillaceae abundance (Fig. 3f).

### Transplantation of fecal microbiota from EAP mice with HSD elicited more severe prostate inflammation in psuedogerm-free mice

**Fig. 4:**
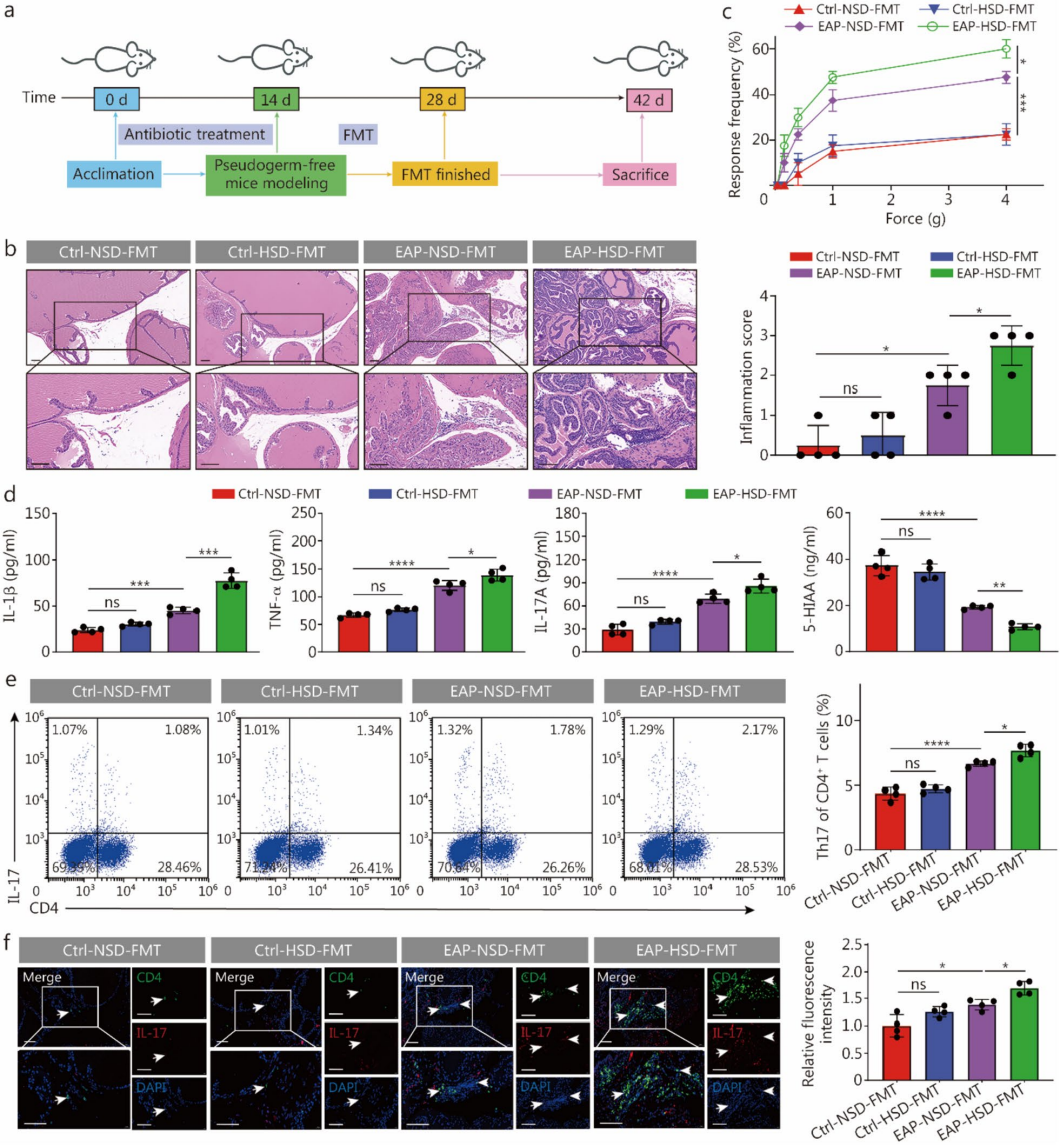
Transplantation of fecal microbiota from EAP mice with HSD elicited more severe prostate inflammation in psuedogerm-free mice. **a** Simple schematic diagram of the experimental workflow. **b** HE staining and inflammation scores of prostate tissues (scale bar = 100 μm, *n* = 4). **c** Development of tactile allodynia in mice belonging to the 4 groups (*n* = 4). **d** The concentrations of IL-1β, TNF-α, IL-17A, and 5-HIAA in the serum of NOD mice in the 4 groups were measured (*n* = 4). **e** Proportion of Th17 of CD4^+^ T cells, as determined by flow cytometry, among the splenic lymphocytes of immunized mice in the 4 groups (*n* = 4). **f** The infiltration of Th17 cells in prostate tissues from mice was determined by immunofluorescence (white arrowheads, scale bar = 100 μm, *n* = 4). ^*^P < 0.05, ^**^P < 0.01, ^***^P < 0.001, ^****^P < 0.0001; ns non-significant. NOD nonobese male diabetic/LtJ, EAP experimental autoimmune prostatitis, HSD high-salt diet, NSD normal-salt diet, FMT fecal microbiota transplantation, HE hematoxylin–eosin, IL-1β interleukin-1β, TNF-α tumor necrosis factor-α, IL-17A interleukin-17A, 5-HIAA 5-hydroxyindole acetic acid, DAPI 4',6-diamidino-2-phenylindole

To determine whether alterations in gut microbiota are responsible for the exacerbating effects of HSD on CP/CPPS, we conducted FMT experiments by administering fecal suspensions from Ctrl + NSD, Ctrl + HSD, EAP + NSD, and EAP + HSD mice to antibiotics-treated pseudogerm-free recipient mice, respectively (Fig. 4a). The prostate inflammation in mice that received fecal suspensions from the EAP + HSD group was significantly more severe than those in mice that received fecal suspensions from the EAP + NSD group (Fig. 4b). Similarly, the frequency of tactile allodynia was markedly greater at forces of 1.0 and 4.0 g in the mice that received fecal suspensions from the EAP + HSD group than those in mice that received fecal suspensions from the EAP + NSD group (Fig. 4c). Furthermore, the concentrations of IL-1β, TNF-α, and IL-17A in the serum of mice that received fecal suspensions from the EAP + HSD group were markedly greater than those in the serum of mice that received fecal suspensions from the EAP + NSD group (Fig. 4d). The decrease in 5-HIAA abundance contributed significantly to the emergence of EAP mice fed an HSD, as shown by the findings of the integrated association analysis of 16S rRNA sequencing and untargeted metabolomics data and by the enriched pathways identified in the KEGG analysis. Therefore, we measured the content of 5-HIAA in the serum samples of the mice subjected to FMT. Compared with the EAP + NSD group, the EAP + HSD group presented far lower levels of 5-HIAA (Fig. 4d). Given that HSD promoted the differentiation of Th17 cells in EAP mice, we next assessed the differentiation of CD4^+^ T cells to Th17 cells in FMT-treated mice. These findings revealed that, compared to mice receiving an FMT from the EAP + NSD group, mice receiving an FMT from the EAP + HSD group had a greater percentage of Th17 cells (Fig. 4e). Furthermore, compared with the mice that received FMT from the EAP + NSD group, the mice that received FMT from the EAP + HSD group presented increased infiltration of Th17 cells into prostate tissues (Fig. 4f). The findings shown in Fig. 4 indicate that the alteration of the gut microbiota was responsible for the exacerbating role of HSD on CP/CPPS by promoting the differentiation of CD4^+^ T cells to Th17 cells.

### 5-HIAA supplementation ameliorated the symptoms of EAP caused by HSD

**Fig. 5:**
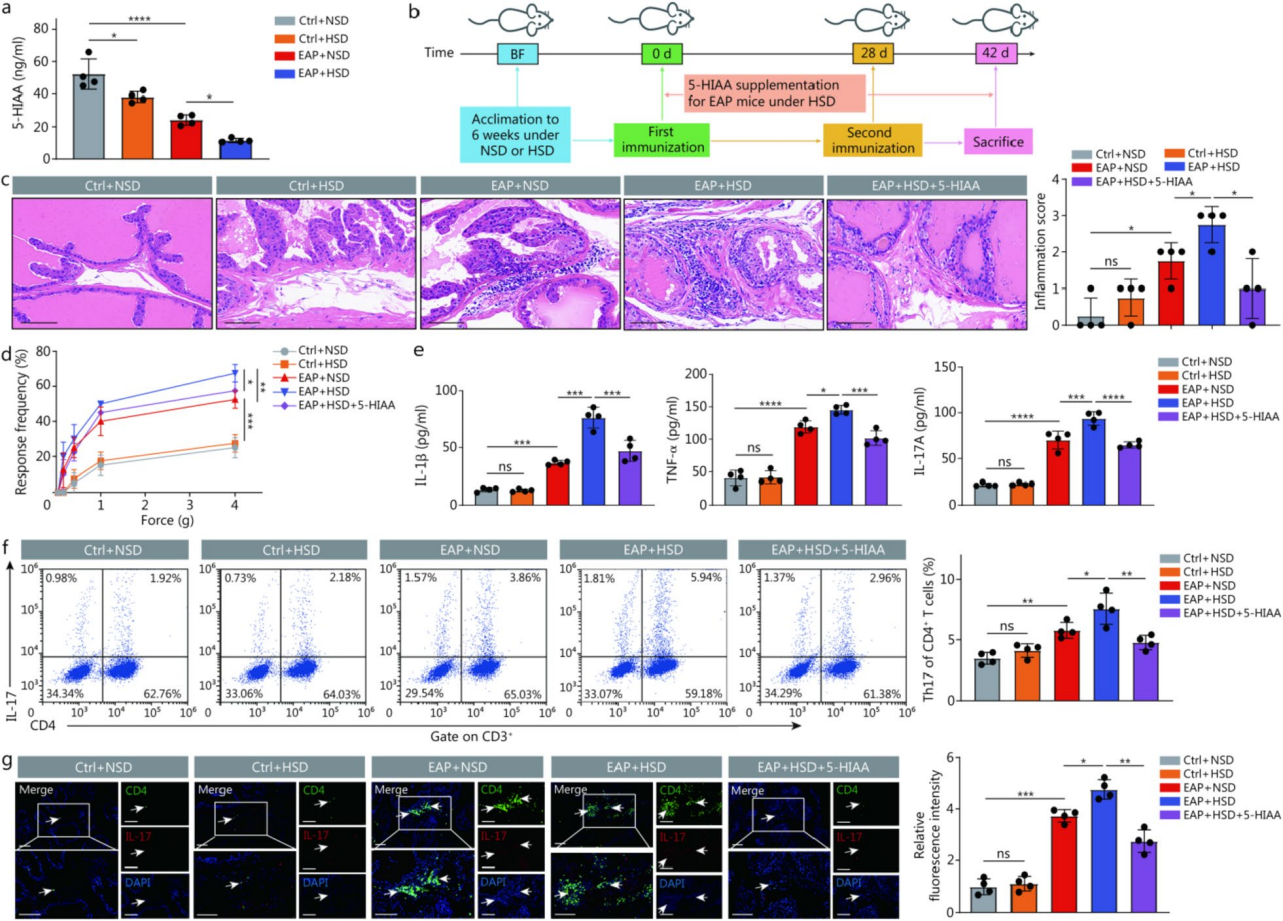
5-HIAA supplementation ameliorated the symptoms of EAP caused by HSD. **a** The levels of 5-HIAA secreted into the serum of the mice were measured in 4 groups: Ctrl + NSD, Ctrl + HSD, EAP + NSD, and EAP + HSD groups (*n* = 4). **b** Simple schematic diagram of the experimental workflow. **c** HE staining and inflammation scores of prostate tissues (scale bar = 100 μm, *n* = 4). **d** Tactile allodynia development in NOD mice in the 4 groups (*n* = 4). **e** Secretion levels of IL-1β, TNF-α, and IL-17A in the serum of mice in the 5 groups (*n* = 4). **f** Flow cytometry was used to determine the proportion of Th17 of CD4^+^ T cells among the splenic lymphocytes of immunized mice in the 5 groups (*n* = 4).** g** The infiltration of Th17 cells in prostate tissues from mice was determined by immunofluorescence (white arrowheads, scale bar = 100 μm, *n* = 4). ^*^P < 0.05, ^**^P < 0.01, ^***^P < 0.001, ^****^P < 0.0001; ns non-significant. NOD nonobese male diabetic/LtJ, BF breeding feed, EAP experimental autoimmune prostatitis, HSD high-salt diet, NSD normal-salt diet, HE hematoxylin–eosin, IL-1β interleukin-1β, TNF-α tumor necrosis factor-α, IL-17A interleukin-17A, 5-HIAA 5-hydroxyindole acetic acid, DAPI 4',6-diamidino-2-phenylindole

To further explore whether the decrease in 5-HIAA abundance is linked to the exacerbating effect of HSD on EAP and the potential therapeutic effect of 5-HIAA supplementation, we assessed the contents of 5-HIAA in the serum samples of normal or EAP mode mice fed an HSD or not (Fig. 5a), some of which were supplemented with 5-HIAA (Fig. 5b). The levels of 5-HIAA in the EAP + HSD mice was lower than that in the EAP + NSD mice (Fig. 5a). Supplementation with 5-HIAA significantly reduced prostate inflammation and pelvic pain in EAP + HSD mice (Fig. 5c, d). Furthermore, the serum IL-1β, TNF-α, and IL-17A levels in the EAP + HSD mice were markedly reduced upon supplementation with 5-HIAA (Fig. 5e). Surprisingly, compared with that in the mice not receiving 5-HIAA supplementation, the percentage of Th17 cells in the EAP + HSD mice was lower following the administration of 5-HIAA (Fig. 5f). Moreover, compared with that in the prostate tissues of the mice not receiving 5-HIAA supplementation, the infiltration of Th17 cells in the prostate tissues of the EAP + HSD mice was reduced following the administration of 5-HIAA (Fig. 5g). These results demonstrated that the decrease in 5-HIAA abundance was responsible for the exacerbating effects of HSD on EAP by promoting the differentiation of CD4^+^ T cells to Th17 cells.

### Abrogation of the protective effects of 5-HIAA supplementation on EAP during HSD was accomplished by CH-mediated inhibition of AHR

**Fig. 6:**
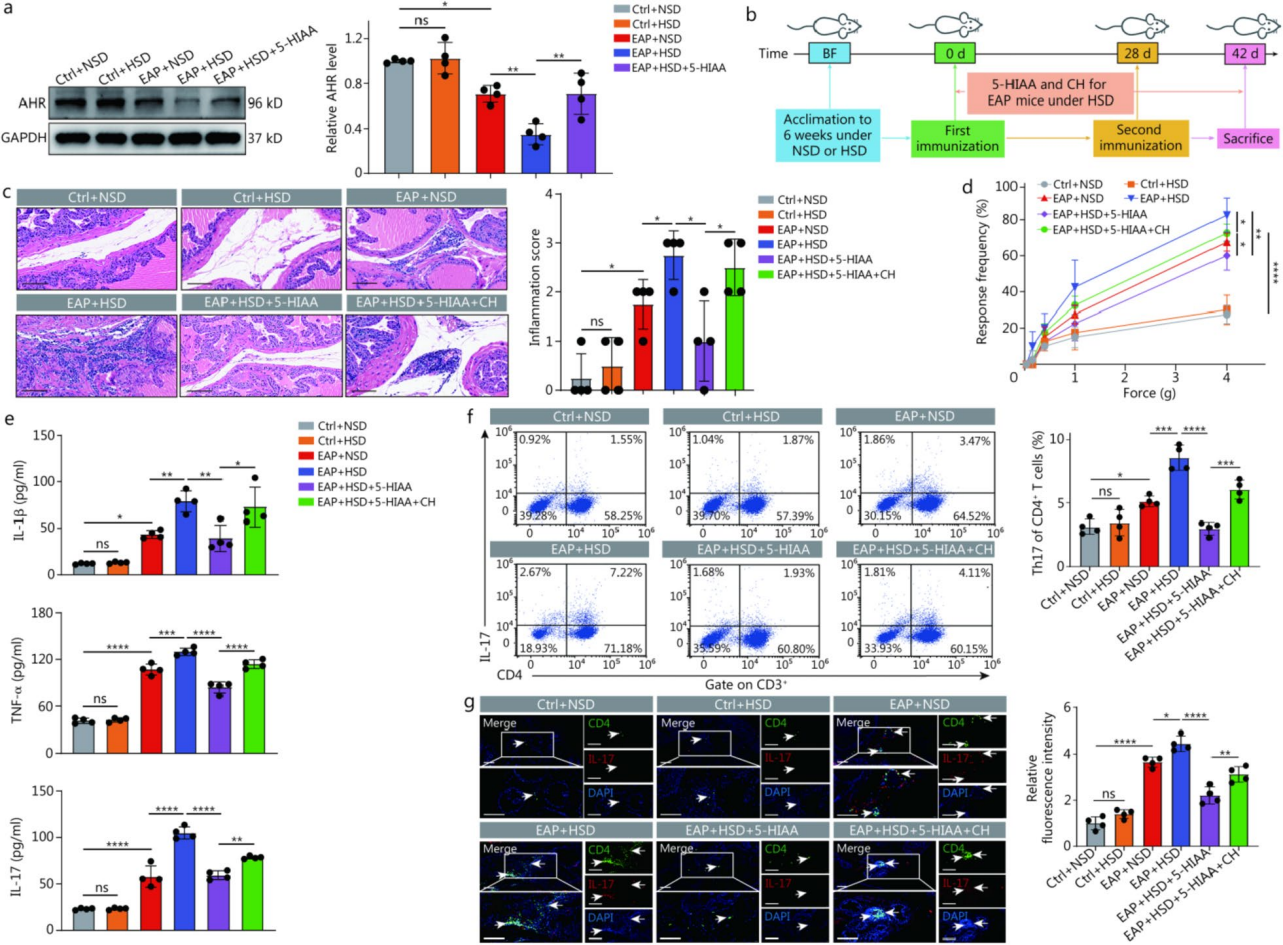
Inhibition of AHR abrogated the protective effects of 5-HIAA supplementation on EAP mice fed an HSD. **a** AHR levels in prostate tissues of mice among the Ctrl + NSD, Ctrl + HSD, EAP + NSD, EAP + HSD, and EAP + HSD + 5-HIAA groups (*n* = 4). **b** A simple schematic diagram of the experimental workflow. **c** HE staining and inflammation scores of prostate tissues (scale bar = 100 μm, *n* = 4). **d** Tactile allodynia development in the mice from the Ctrl + NSD, Ctrl + HSD, EAP + NSD, EAP + HSD, EAP + HSD + 5-HIAA, and EAP + HSD + 5-HIAA + CH groups (*n* = 4). **e** The levels of IL-1β, TNF-α, and IL-17A in the serum of the mice in the 6 groups (*n* = 4). **f** Proportion of Th17 of CD4^+^ T cells, as determined by flow cytometry, among the splenic lymphocytes of immunized mice in the 6 groups (white arrowheads, *n* = 4). **g** The infiltration of Th17 cells in prostate tissues from mice was evaluated by immunofluorescence (scale bar = 100 μm, *n* = 4). ^*^P < 0.05, ^**^P < 0.01, ^***^P < 0.001, ^****^P < 0.0001; ns non-significant. NOD nonobese male diabetic/LtJ, BF breeding feed, EAP experimental autoimmune prostatitis, HSD high-salt diet, NSD normal-salt diet, CH CH223191 (an AHR inhibitor), HE hematoxylin–eosin, IL-1β interleukin-1β, TNF-α tumor necrosis factor-α, IL-17A interleukin-17A, 5-HIAA 5-hydroxyindole acetic acid, AHR aryl hydrocarbon receptor, DAPI 4',6-diamidino-2-phenylindole

As a derivative of indoleacetic acid, 5-HIAA generally activates the AHR to regulate the immune response [25]. Thus, we investigated whether the protective effect of 5-HIAA supplementation in EAP + HSD mice was mediated by promoting the activation of AHR. AHR levels were decreased in EAP + HSD mice, whereas this effect was abrogated after supplementation with 5-HIAA (Fig. 6a). We intraperitoneally injected the AHR inhibitor CH followed by 5-HIAA supplementation to subsequently confirm that the protective effect of 5-HIAA supplementation in EAP + HSD mice was dependent on AHR activity (Fig. 6b). Compared with the mice from EAP + HSD + 5-HIAA group, mice from EAP + HSD + 5-HIAA + CH group presented a resurgence of prostate inflammation (Fig. 6c). Similarly, compared with that in the EAP + HSD + 5-HIAA group, pelvic pain in the EAP + HSD + 5-HIAA + CH group was exacerbated at forces of 1.0 and 4.0 g (Fig. 6d). Compared with the EAP + HSD + 5-HIAA group, the EAP + HSD + 5-HIAA + CH group presented significantly higher IL-1β, TNF-α, and IL-17A levels (Fig. 6e). In addition, the percentage of Th17 cells was greater in the EAP + HSD + 5-HIAA + CH group than in the EAP + HSD + 5-HIAA group (Fig. 6f). Moreover, the infiltration of Th17 cells was greater in the prostate tissues of mice from EAP + HSD + 5-HIAA + CH group than those of mice from EAP + HSD + 5-HIAA group (Fig. 6g). These results indicated that the protective influence of 5-HIAA supplementation in EAP + HSD mice was contingent upon the stimulation of AHR, which subsequently facilitated the differentiation of CD4^+^ T cells to Th17 cells.

### HSD promoted the differentiation of Th17 cells by inhibiting the 5-HIAA/AHR axis to activate the SGK1/FOXO1 signaling pathway

**Fig. 7:**
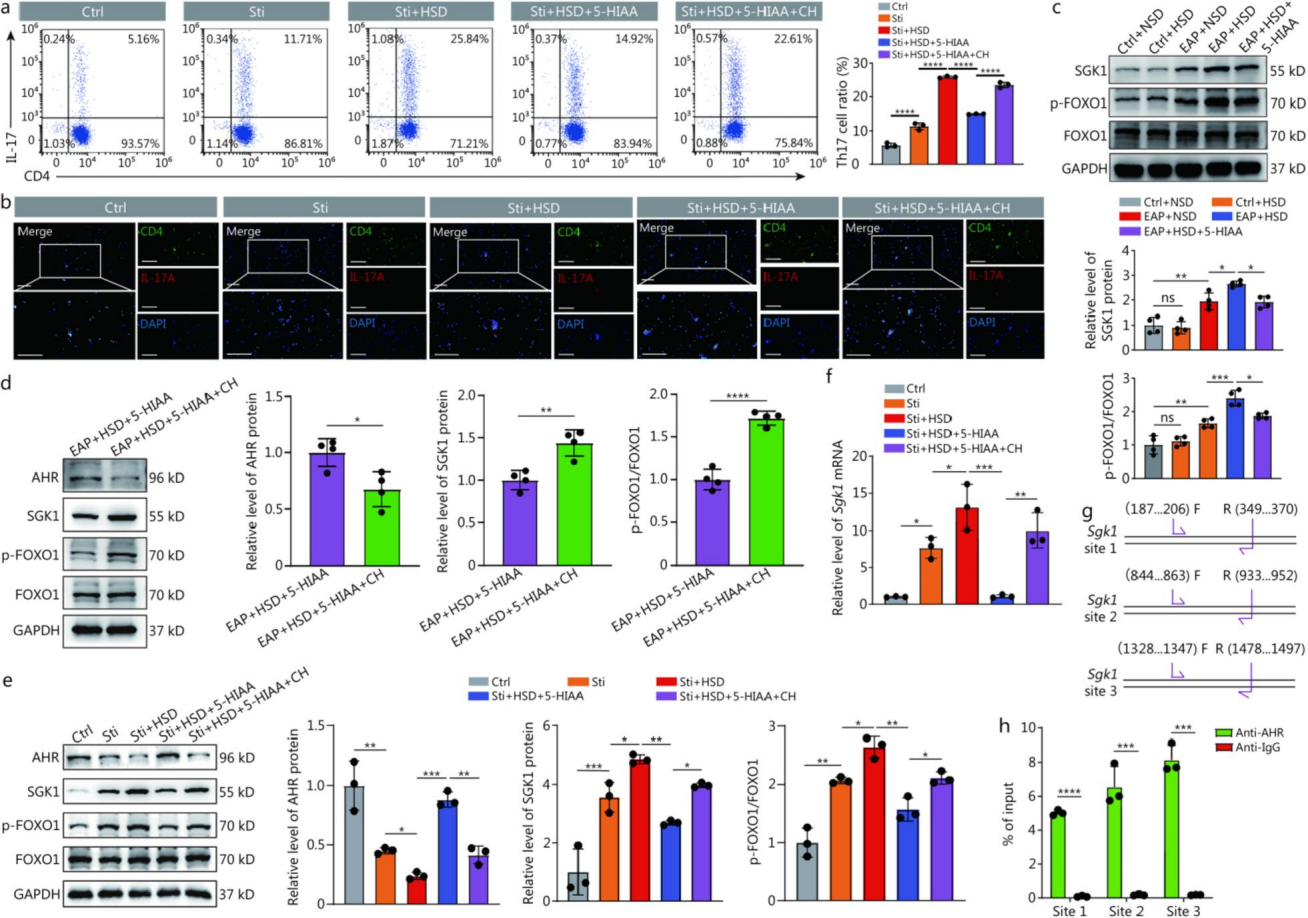
HSD promoted the differentiation of Th17 cells by activating SGK1/FOXO1 signaling pathway. **a** The in vitro Th17 cell differentiation ratio was determined by flow cytometry (*n* = 3). **b** Th17 cell differentiation ratio was determined in an in vitro differentiation experiment using immunofluorescence (scale bar = 100 μm, *n* = 3). **c** SGK1, p-FOXO1, and FOXO1 levels in prostate tissues from Ctrl + NSD, Ctrl + HSD, EAP + NSD, EAP + HSD, and EAP + HSD + 5-HIAA mice (*n* = 4). **d** AHR, SGK1, p-FOXO1, and FOXO1 levels in prostate tissues of mice in the EAP + HSD + 5-HIAA and EAP + HSD + 5-HIAA + CH groups (*n* = 4). **e** AHR, SGK1, p-FOXO1, and FOXO1 levels in naïve CD4^+^ T cells of Ctrl, Sti, Sti + HSD, Sti + HSD + 5-HIAA, Sti + HSD + 5-HIAA + CH groups (*n* = 3). **f** Transcriptional levels of *Sgk1* in naïve CD4^+^ T cells of Ctrl, Sti, Sti + HSD, Sti + HSD + 5-HIAA, Sti + HSD + 5-HIAA + CH groups (n = 3). **g** For the ChIP-qPCR assay, 3 potential enhancer segments of the mouse promoter region of *Sgk1* were created. **h** qPCR was used to assess the immunoprecipitated DNA by a ChIP assay (*n* = 3). ^*^P < 0.05, ^**^P < 0.01, ^***^P < 0.001, ^****^P < 0.0001; ns non-significant. EAP experimental autoimmune prostatitis, HSD high-salt diet, NSD normal-salt diet, IL-17A interleukin-17A, 5-HIAA 5-hydroxyindole acetic acid, AHR aryl hydrocarbon receptor, SGK1 serum and glucocorticoid-regulated kinase 1, FOXO1 forkhead box protein O1, CH CH223191 (an AHR inhibitor), ChIP chromatin immunoprecipitation, qPCR quantitative polymerase chain reaction, DAPI 4’,6-diamidino-2-phenylindole, Sti stimulation with IL-6, IL-23, TGF-β, anti-IFN-γ, and anti-IL-4

Next, we isolated naïve CD4^+^ T cells for differentiation induction to investigate the effect of 5-HIAA on Th17 cell differentiation, and the results revealed that 5-HIAA inhibited the differentiation of CD4^+^ T cells to Th17 cells in vitro (Additional file 1: Fig. S1a). To further investigate the underlying mechanism by which HSD promoted Th17 cell differentiation, we detected Th17 cell differentiation under HSD stimulation or not and with or without 5-HIAA and CH. HSD stimulation promoted the transformation of naïve CD4^+^ T cells to Th17 cells, as predicted. In contrast, 5-HIAA impeded the differentiation of naïve CD4^+^ T cells to Th17 cells. CH counteracted the inhibitory impact of 5-HIAA on the process of differentiating naïve CD4^+^ T cells into Th17 cells (Fig. 7a, b). Subsequently, we examined the effects of a high-salt treatment on the differentiation of human naïve CD4^+^ T cells and found that alterations in the transformation of human naïve CD4^+^ T cells into Th17 cells under HSD stimulation with or without 5-HIAA and CH mirrored the results obtained in the mouse model (Additional file 1: Fig. S1b). Furthermore, during Th17 cell differentiation in vitro, the RORγt levels of naïve CD4^+^ T cells were consistent with the changes observed in the differentiation ratios of Th17 cells from naïve CD4^+^ T cells, after HSD stimulation or not and with or without 5-HIAA and CH. HSD stimulation increased RORγt levels, whereas supplementation with 5-HIAA decreased the levels of RORγt. In contrast, the administration of CH abrogated the regulatory effects of 5-HIAA (Additional file 1: Fig. S1c). Research has demonstrated that SGK1, an enzyme involved in maintaining sodium balance, is necessary for maintaining Th17 cell phenotypes. This is achieved by inactivating forkhead box protein O1 (FOXO1) through phosphorylation, which leads to its relocation from the nucleus to the cytoplasm [26, 27]. Therefore, we next determined whether HSD promoted the differentiation of Th17 cells by inhibiting the 5-HIAA/AHR axis and activating the SGK1/FOXO1 signaling pathway. SGK1 and phosphorylated FOXO1 were expressed at significantly higher levels in EAP + HSD mice than in EAP + NSD mice. Nevertheless, following the administration of 5-HIAA, there was a notable decrease in the levels of SGK1 and phosphorylated FOXO1 (Fig. 7c). Furthermore, the suppression of AHR with CH restored the levels of SGK1 and phosphorylated FOXO1 (Fig. 7d). Consistent with these findings, during the differentiation of CD4^+^ T cells to Th17 cells in vitro, the levels of AHR decreased while the levels of SGK1 and phosphorylated FOXO1 increased in naïve CD4^+^ T cells after HSD stimulation. Notably, supplementation with 5-HIAA restored the levels of AHR, and SGK1, and phosphorylated FOXO1 to control normal levels, whereas the administration of CH partially abrogated the regulatory effects of 5-HIAA (Fig. 7e).

**Fig. 8:**
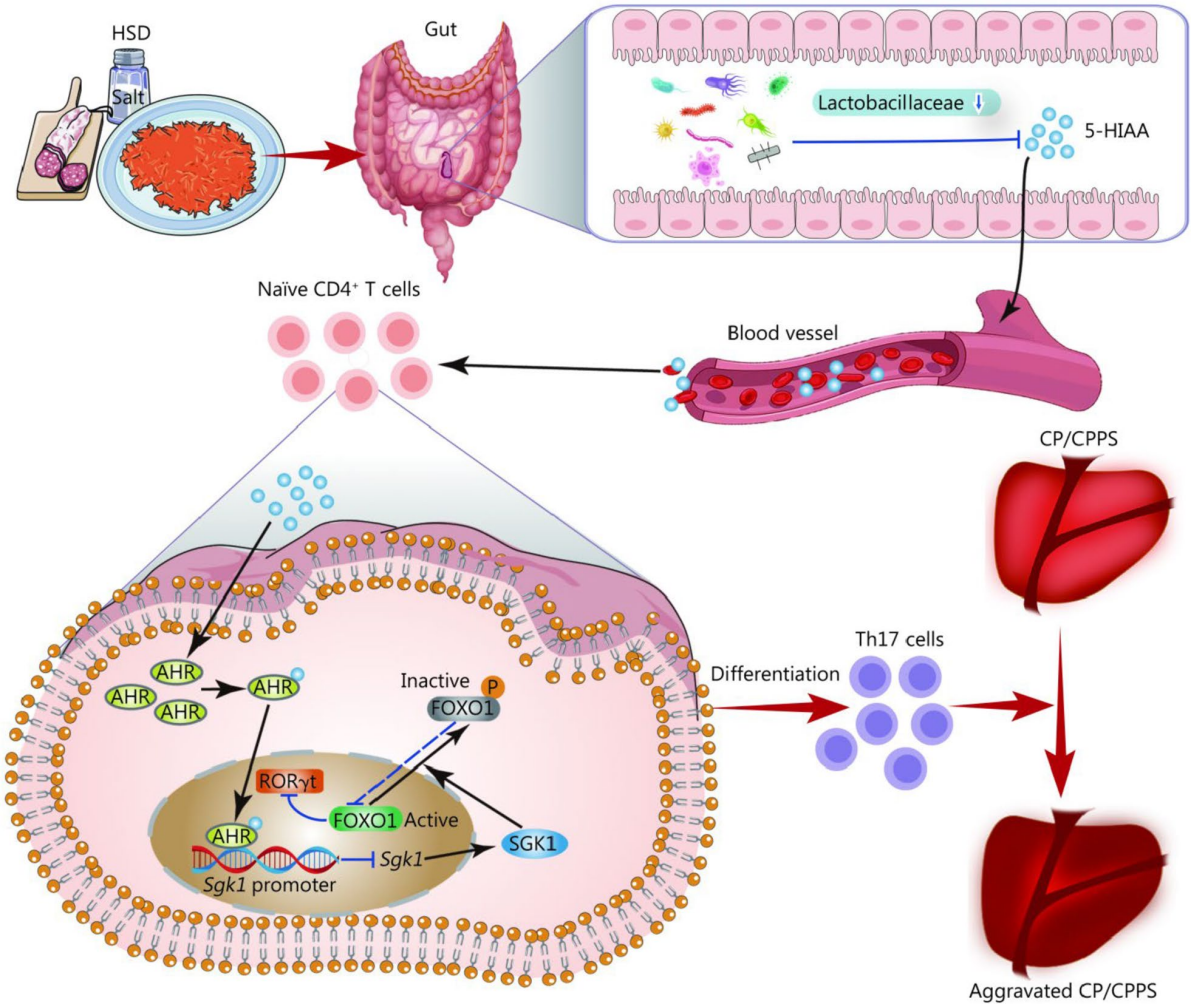
Schematic diagram illustrating the function and mechanism of HSD in the progression of CP/CPPS. HSD elevated the levels of SGK1 by inhibiting the 5-HIAA/AHR axis and further induced the phosphorylation of FOXO1, consequently promoting RORγt-driven Th17 cell differentiation to exacerbate CP/CPPS. HSD high-salt diet, CP/CPPS chronic prostatitis/chronic pelvic pain syndrome, 5-HIAA 5-hydroxyindole acetic acid, AHR aryl hydrocarbon receptor, SGK1 serum and glucocorticoid-regulated kinase 1, FOXO1 forkhead box protein O1, RORγt retinoic acid receptor-related orphan receptor gamma-t

The nuclear AHR regulates responses to extracellular stimuli. It can also modulate disease, particularly by regulating inflammatory and immune responses, as reflected by the presence of AHR binding sequence sites on the promoters or enhancers of several inflammatory cytokines, receptors, and functional proteins [28]. On the basis of the aforementioned findings, we hypothesized that the 5-HIAA/AHR axis may negatively regulate *Sgk1* transcription, which has not been reported thus far. In this study, during the differentiation of CD4^+^ T cells to Th17 cells in vitro, the *Sgk1* transcription levels increased in naïve CD4^+^ T cells after HSD stimulation, while supplementation with 5-HIAA decreased the transcriptional levels of *Sgk1*, which can be partially reversed by the administration of CH (Fig. 7f). In addition, the sequences of the AHR binding sites on the *Sgk1* promoter were predicted by using JASPAR matrix models. For the ChIP-qPCR assay, three putative fragments of the promoter region of *Sgk1* were designed (Fig. 7g). As a result, the ChIP assay verified the interaction of the AHR protein with the *Sgk1* DNA fragment in CD4^+^ T cells, and the immunoprecipitated DNA was detected via qPCR (Fig. 7h). These findings suggested that AHR might function as a transcription factor by interacting with the *Sgk1* promoter in CD4^+^ T cells. Collectively, the results shown in Fig. 7 demonstrated that HSD potentially increased the expression of SGK1, further inducing the phosphorylation of FOXO1 and causing increased the differentiation of CD4^+^ T cells to Th17 cells via the inhibition of the 5-HIAA/AHR axis. Targeting the recently discovered 5-HIAA/AHR/SGK1/FOXO1 pathway could offer innovative treatment options for patients with CP/CPPS (Fig. 8).

## Discussion

CP/CPPS is a common genitourinary disease in men. Nevertheless, existing therapeutic approaches have failed to achieve desirable clinical efficacy because of the complicated nature of the disease. Therefore, it is essential to perform comprehensive investigations to identify the fundamental mechanism and risk factors of CP/CPPS to increase the efficacy of clinical therapies. The present study revealed that HSD can increase inflammation and pain symptoms in EAP mice by boosting the differentiation of CD4^+^ T cells to Th17 cells. Moreover, HSD reduced the abundance of Lactobacillaceae and Desulfovibrionaceae, as well as the content of 5-HIAA, which is related to tryptophan metabolism. This alteration promoted the expression of SGK1, leading to increased phosphorylation of FOXO1 and the differentiation of Th17 cells.

Although genetics and gender are both involved in the etiology of various autoimmune diseases, the contribution of environmental factors should not be ignored [29]. For instance, the ionic composition of the local microenvironment is a pivotal determinant of tissue-specific T-cell effector responses under both physiological and pathological conditions [30, 31], and excessive intake of NaCl may increase the risk of autoimmune encephalomyelitis, ulcerative colitis [32, 33]. Previously, there was limited knowledge regarding the impact of HSD on CP/CPPS, while our present findings indicated that patients with CP/CPPS had significantly greater sodium intake, more severe prostate inflammation, and higher pain sensitivity were documented in mice fed an HSD compared with mice fed an NSD.

Of note, the association between HSD-related diseases and the differentiation of CD4^+^ T cells to proinflammatory Th1 and Th17 cells has been explored [34]. Furthermore, proinflammatory Th1 and Th17 cells have been revealed to play crucial roles in the autoimmune responses of CP/CPPS [9, 10]. Herein, we revealed that HSD stimulated the differentiation of CD4^+^ T cells to Th17 cells in EAP mice, while there was no substantial disparity in the ratio of Th1 cells between EAP mice fed an NSD or an HSD. Thus, we hypothesized that HSD exacerbated EAP by facilitating the differentiation of CD4^+^ T cells to Th17 cells.

The gut microbiota has been linked to the onset and progression of several diseases, such as inflammatory bowel diseases, colon cancer, autism, and Parkinson’s disease, and the diversity and composition of microbiota communities may be influenced by dietary habits [35–37]. Especially, it is well-documented that HSD can influence the gut microbiota composition, potentially leading to various inflammatory diseases [38]. However, it is not known how HSD affects the composition and function of the gut microbiota, let alone whether changes in the microbiota are involved in the development of CP/CPPS. Therefore, we analyzed the dynamics of the fecal microbiota composition and changes in metabolite abundance following HSD consumption and documented the function and altered gut microbiota composition. Specifically, the relative abundances of Lactobacillaceae and Desulfovibrionaceae, as well as the tryptophan metabolite 5-HIAA, decreased significantly. Subsequent FMT and 5-HIAA supplementation experiments verified that HSD consumption changed the microbiome composition by lowering 5-HIAA levels, which in turn caused Th17 cells to differentiate in a dysregulated manner and CP/CPPS to progress.

Multiple biological processes are modulated by the cytosolic ligand-activated transcription factor AHR. Furthermore, it is a crucial immunological response regulator that modulates the differentiation of Th17 cells. Thus, the activation of AHR has been reported to be involved in disease progression and antibacterial defense in multiple preclinical investigations [39]. Tryptophan metabolites, including indoleacetic acid, kynurenine, and 5-HIAA, are important endogenous ligands of AHR. 5-HIAA can activate AHR-dependent gene transcription, exerting a significant immunoregulatory effect in the context of various diseases [25]. Nevertheless, it remains uncertain whether 5-HIAA exacerbates CP/CPPS by stimulating AHR. In the present study, an AHR inhibitor (CH) was able to abrogate the protective effect of 5-HIAA supplementation in EAP mice fed an HSD. This abrogation occurred through an increase in the differentiation of Th17 cells. These results indicate that 5-HIAA supplementation exerts its therapeutic effects on EAP mice fed an HSD through AHR activation and subsequent Th17 cell differentiation.

SGK1 can be activated by exogenous NaCl and generally functions as a mediator of salt homeostasis. SGK1 has been researched in detail concerning NaCl transport because it regulates sodium intake through phosphorylating epithelial sodium channels. In vitro, the differentiation of CD4^+^ T cells to Th17 cells and increased IL-23R expression may result from a small increase in NaCl content triggering SGK1 expression in T cells [40]. Several studies have shown that HSD aggravates autoimmune encephalomyelitis in an SGK1-dependent manner by promoting Th17 cell differentiation [16, 17, 32, 40]. Furthermore, SGK1 is a crucial node downstream of IL-23 that, by deactivating FOXO1, a direct suppressor of IL-23R production, is necessary to stabilize the Th17 cell phenotype [17]. The present study revealed that HSD consumption promoted the differentiation of CD4^+^ T cells to Th17 cells by inhibiting the 5-HIAA/AHR axis and activating the SGK1/FOXO1 signaling pathway. Moreover, AHR can regulate gene transcriptional activity by binding to the xenobiotic response element in the promoter of the target gene [28]. In this study, we found that AHR could act as a transcription factor by binding to the promoter of *Sgk1* in CD4^+^ T cells, which has not been reported previously. In addition, proinflammatory cells can migrate to the site of inflammation and increase the production of reactive oxygen species (ROS), which can be evaluated by assessing the levels of malonaldehyde [41]. Similar to the role of ROS in other diseases [42, 43], excessive production of ROS can lead to the occurrence and development of CP/CPPS [44]. It is worthing that HSD can also exacerbate inflammatory diseases by stimulating the production of ROS [45]. On top of that, nuclear factor-erythroid 2 related factor 2 (Nrf2) is a key transcription factor that regulates the endogenous ROS clearance system [46]. The anti-inflammatory properties of redox-active compounds have been explored, particularly through activating Nrf2 to inhibit oxidative stress [47, 48]. Therefore, targeting the Nrf2 signaling pathway may be an effective therapeutic strategy for CP/CPPS under HSD.

Our study has several limitations. First, although our experiments suggested that HSD reduced the relative abundance of the gut microflora and metabolites related to tryptophan metabolism, specific clinical trials are necessary to validate these findings. Second, the specific function of the 5-HIAA/AHR axis in exacerbating CP/CPPS under HSD has not been verified in *Ahr*-KO mice. Third, although we demonstrated that HSD promoted Th17 cell differentiation by activating the SGK1/FOXO1 pathway via inhibition of the 5-HIAA/AHR axis, the possibility of other unidentified pathways cannot be fully excluded.

## Conclusions

In summary, our study demonstrated that HSD promotes the expression of SGK1 by inhibiting the 5-HIAA/AHR axis and inducing the phosphorylation of FOXO1, which in turn promotes Th17 cell differentiation and exacerbates CP/CPPS. Targeting this newly identified 5-HIAA/AHR/SGK1/FOXO1 axis may provide promising therapeutic options for patients with CP/CPPS.

## Supplementary Information

**Additional file 1: Methods**. **Table S1** Demographic characteristics of the CP/CPPS patients and healthy controls. **Table S2** Gene primers for ChIP-qPCR. **Table S3** Salt consumption levels of CP/CPPS patients with different symptoms. **Fig. S1** HSD promoted the differentiation of Th17 cells.

## Data Availability

All data associated with this study are available from the corresponding author with reasonable request.

## Abbreviations

AHR: Aryl hydrocarbon receptor
APC: Allophycocyanin
CFA: Complete Freund’s adjuvant
ChIP: Chromatin immunoprecipitation
CP/CPPS: Chronic prostatitis/chronic pelvic pain syndrome
DAPI: 4',6-Diamidino-2-phenylindole
EAP: Experimental autoimmune prostatitis
ELISA: Enzyme-linked immunosorbent assay
FMT: Fecal microbiota transplantation
FITC: Fluorescein isothiocyanate
FOXO1: Forkhead box protein O1
HE: Hematoxylin-eosin
HSD: High-salt diet
5-HIAA: 5-Hydroxyindole acetic acid
IFN-γ: Interferon-γ
IL-1β: Interleukin-1β
IL-17A: Interleukin-17A
LC–MS: Liquid chromatography-mass spectrometry
KEGG: Kyoto encyclopedia of genes and genomes
NaCl: Sodium chloride
NIH-CPSI: National institutes of health chronic prostatitis symptom index
NOD: Non-obese male diabetic/LtJ
NSD: Normal-salt diet
Nrf2: Nuclear factor-erythroid 2 related factor 2
PAgs: Prostate antigens
qPCR: Quantitative polymerase chain reaction
RORγt: Retinoic acid receptor-related orphan receptor gamma-t
ROS: Reactive oxygen species
16S rRNA: 16S ribosomal RNA
SGK1: Serum and glucocorticoid-regulated kinase 1
TGF-β1: Transforming growth factor-β1
Th1: T helper 1
Th17: T helper 17
TNF-α: Tumor necrosis factor-α
