# Supplementary material for: High-salt-driven gut microbiota dysfunction aggravates prostatitis by promoting AHR/SGK1/FOXO1 axis-mediated Th17 cell differentiation
Source: Mil Med Res. 2025 May 19;12:21. doi: 10.1186/s40779-025-00607-1 (PMC12087074; doi:10.1186/s40779-025-00607-1)
Supplement: Supplementary file 1 — Additional file 1: Methods. Table S1 Demographic characteristics of the CP/CPPS patients and healthy controls. Table S2 Gene primers for ChIP-qPCR. Table S3 Salt consumption levels of CP/CPPS patients with different symptoms. Fig. S1 HSD promoted the differentiation of Th17 cells. [file 40779_2025_607_MOESM1_ESM.pdf]

Methods

The symptom grading based on the NIH-CPSI score

The severity of symptoms was classified according to the National Institutes of Health Chronic Prostatitis Symptom Index (NIH-CPSI) scores as follows: total NIH-CPSI score: mild ≤ 14 points, moderate ≤ 29 points, and severe ≥ 30 points; pain or discomfort score: mild ≤ 7 points, moderate ≤ 14 points, and severe ≥ 14 points; urination score: mild ≤ 3 points, moderate ≤ 6 points, and severe ≥ 7 points; quality of life score: mild ≤ 4 points, moderate ≤ 9 points, and severe ≥ 10 points. For analytical purposes, moderate and severe cases were consolidated into a single category.

16S ribosomal RNA (16S rRNA) sequencing and DNA extraction

Mice cecum content samples were treated to isolate microbial genomic DNA using the E.Z.N.A.® DNA kit (#R6731-01, Omega Bio-Tek, USA). The highly variable V3 – V4 regions of the bacterial 16S rRNA gene were amplified 27 times using polymerase chain reaction (PCR). The Illumina MiSeqPE300 platform was utilized for the measurement and sequencing of the refined PCR products. FLASH (version 1.2.7) was used for splicing, and fastp (version 0.20.0) was used for quality checking the raw sequences used for microbiome analysis. Diversity analysis was used to assess the richness and diversity of the samples. The results were then subjected to the Wilcoxon rank sum test between the exponential groups. A distance visualization tool called principal coordinate analysis was used to analyze the β-diversity of the microbial community. Colony bar plots at the family level and Wilcoxon rank-sum tests of substantially different colonies were used to analyze the variations in species composition between groups. PICRUSt2 was used to predict how high-salt diet (HSD) would affect the fecal microbiota function in experimental autoimmune prostatitis (EAP) model mice.

Metabolome sequencing of serum

To prepare 40 μl aliquots of mouse serum for analysis, an equal volume of 0.15% formic acid was added, and the proteins were precipitated by adding 120 μl of acetonitrile. For optimal extraction, the samples were sonicated for 15 min and agitated at 1400 r/min for 30 min at 4 °C. The samples were centrifuged at 14,000 r/min for 30 min at 4 °C, and the resulting supernatants were transferred to vials. The samples were centrifuged at 14,000 r/min for 30 min at 4 °C, and the resulting supernatants were transferred to vials. The samples were analyzed using a UPLC system coupled with a time-of-flight mass spectrometer. An amide column with dimensions of 2.1 mm × 100 mm and a particle size of 1.7

μm was employed to separate the analytes before mass spectrometry analysis. The mass spectrometer was operated using positive electrospray ionization in full-scan mode. Deviations in the lock mass were automatically corrected for spectral peaks.

Kyoto Encyclopedia of Genes and Genomes (KEGG) database

The KEGG database is designed to understand the functions and interactions of genes, proteins, and metabolites in biological systems such as cells, tissues, and so on. Information on metabolite-related metabolic pathways, human diseases, and drug discovery can be found. Metabolites and metabolic pathways in this database cover two broad categories: eukaryotes (animals, plants, fungi, and protists) and prokaryotes.

Western blotting analysis

Total proteins from cells and prostate tissues were extracted with protease inhibitor-supplemented RIPA buffer (#20120ES60, Yeasen Biotechnology, China). The supernatant was collected after the mixture was centrifuged at 4 °C. The obtained protein sample was denatured in SDS-PAGE loading buffer by immersion in boiling water. After being electrophoresed in a sodium dodecyl sulfate-polyacrylamide gel, the denatured samples were subsequently transferred onto a nitrocellulose membrane via the semidry technique. Nonspecific binding was blocked with a 5% skim milk solution (w/v). The membrane was then incubated with the appropriate primary antibody for 24 h at 4 °C. This was followed by incubation with an HRP-conjugated secondary antibody (1:10000, #S0001, Affinity, USA) for 2 h and subsequent washing in TBST. An enhanced chemiluminescence reagent (#20-500-120, Biological Industries, Israel) was used to visualize the signals on the protein blots, which were then analyzed with ImageJ (version 1.43).

Table S1 Demographic characteristics of the CP/CPPS patients and healthy controls

**Characteristic**	**Healthy control (n = 710)**	**CP/CPPS (n = 434)**	***P*-value**
Age (year, mean ± SD)	24.5 ± 6.9	25.2 ± 7.7	0.1537
Height (cm, mean ± SD)	175.43 ± 5.52	175.26 ± 5.49	0.6168
Weight (kg, mean ± SD)	70.00 ± 11.06	70.95 ± 11.19	0.1633
BMI (kg/m^2, mean ± SD)	22.73 ± 3.30	23.09 ± 3.44	0.0771
Na (mg/d, mean ± SD)	702.04 ± 586.1	783.15 ± 634.10	0.0281
NIH-CPSI score [M (Q_1, Q_3)]	1 (0, 2)	9 (7, 17)	-
Pain or discomfort score [M (Q_1, Q_3)]	0 (0, 0)	3 (0, 7)	-
Urination score [M (Q_1, Q_3)]	1 (0, 1)	2 (1, 4)	-
Quality of life score [M (Q_1, Q_3)]	0 (0, 0)	5 (3, 8)	-

CP/CPPS chronic prostatitis/chronic pelvic pain syndrome, *BMI* body mass index, *NIH-CPSI* National Institutes of Health Chronic Prostatitis Symptom Index

Table S2 Gene primers for ChIP-qPCR

**Gene symbol**	**Forward primer (5' – 3')**	**Reverse primer (5' – 3')**
Sgk1 site 1	GGAGAATCTGCCAAAGCAAG	AAGCCATTCAATCTCTTTCCTG
Sgk1 site 2	GGGTTCTAATCCCATGTTGC	GTGCTTGCATGAAACCATGA
Sgk1 site 3	CAGGATAAGTTGGGGCATGT	AACAGAGAAGCCGGCAGTAA
Gapdh	TCCTTAGCCCTGAGCTGTGT	ATGTTTTCTGGGGTGCAAAG

ChIP chromatin immunoprecipitation, *qPCR* quantitative polymerase chain reaction, *Sgk1* serum and glucocorticoid-regulated kinase 1, *Gapdh* glyceraldehyde-3-phosphate dehydrogenase

Table S3 Salt consumption levels of CP/CPPS patients with different symptoms

**Characteristic**	**Na (mg/d, mean ± SD)**	***P*-value**
NIH-CPSI score		< 0.0001
Mild	643.82 ± 401.26	
Moderate to severe	1127.55 ± 916.21	
Quality of life score		0.0004
Mild	645.01 ± 381.30	
Moderate to severe	867.05 ± 736.15	
Urination score		< 0.0001
Mild	686.00 ± 448.67	
Moderate to severe	1037.35 ± 920.26	
Pain or discomfort score		< 0.0001
Mild	651.60 ± 396.80	
Moderate to severe	1307.82 ± 1023.40	

CP/CPPS chronic prostatitis/chronic pelvic pain syndrome, *NIH-CPSI* National Institutes of Health Chronic Prostatitis Symptom Index

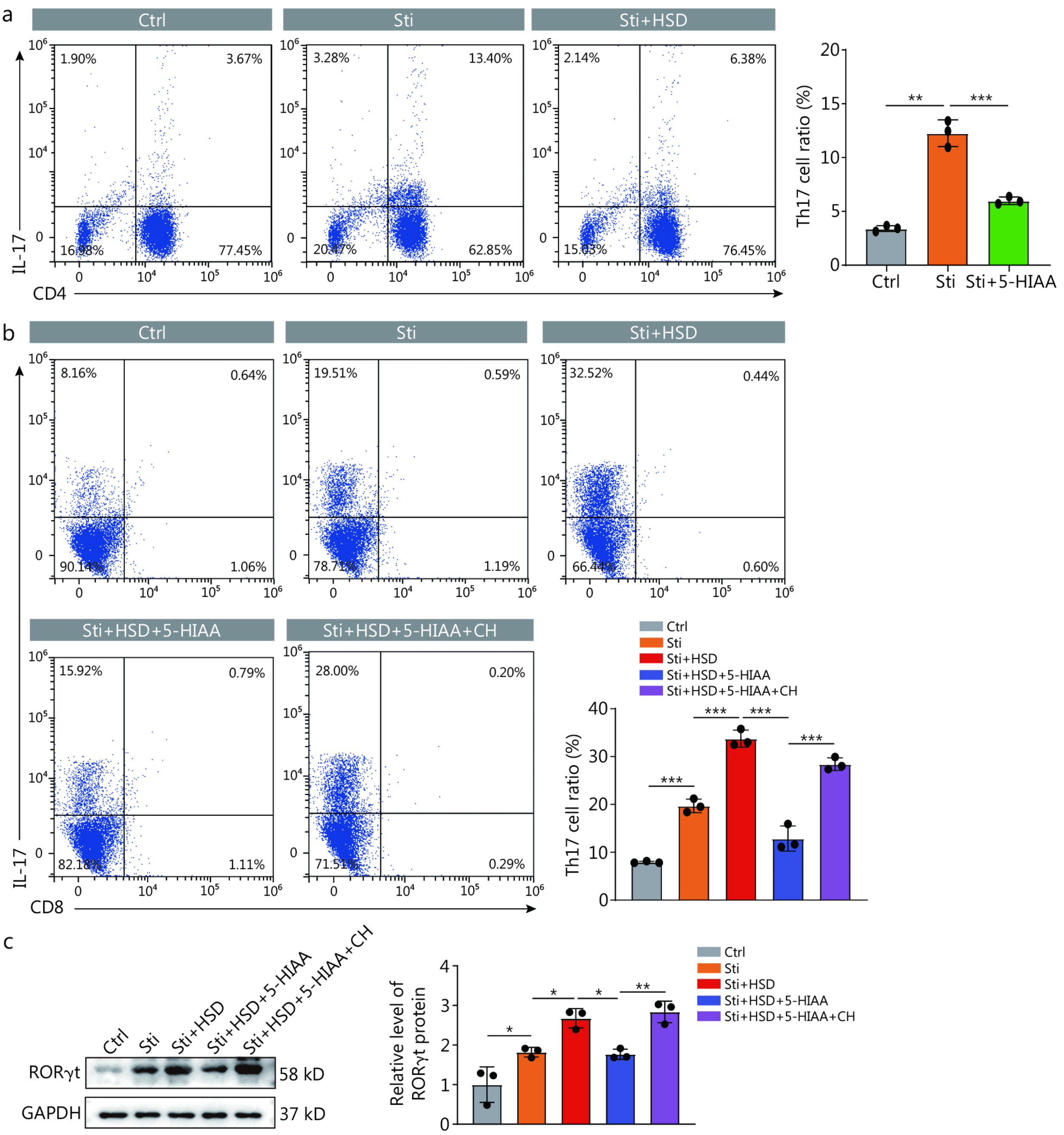

Fig. S1 HSD promoted the differentiation of Th17 cells. **a** The Th17 cell differentiation ratio of naïve $CD4^+$ T cells with or without 5-HIAA was determined by flow cytometry through in vitro assay ($n = 3$). **b** The Th17 cell differentiation ratio of human naïve $CD4^+$ T cells with or without HSD, 5-HIAA, and CH was determined by flow cytometry through in vitro assay ($n = 3$). **c** RORγt levels in naïve $CD4^+$ T cells of Ctrl, Sti, Sti + HSD, Sti + HSD + 5-HIAA, Sti + HSD + 5-HIAA + CH groups ($n = 3$). $^{*}P < 0.05$, $^{**}P < 0.01$, $^{***}P < 0.001$. HSD high-salt diet, NSD normal salt diet, 5-HIAA 5-hydroxyindole acetic acid, RORγt Retinoic acid receptor-related orphan receptor gamma-t, CH CH223191 (an AHR inhibitor), Sti stimulation with IL-6, IL-23, TGF-β, anti-IFN-γ, and anti-IL-4
